# Evaluation of tracheal diameter and angles in fetuses with double aortic arch using prenatal ultrasound: implications for postnatal management

**DOI:** 10.3389/fmed.2024.1398623

**Published:** 2024-08-30

**Authors:** Caihong Jiang, Wen Ling, Longzhuang Peng, Shan Guo, Qiumei Wu, Chunxia Chen, Fa Chen, He Li, Zongjie Weng

**Affiliations:** ^1^Department of Medical Ultrasonics, Fujian Maternity and Child Health Hospital, College of Clinical Medicine for Obstetrics and Gynecology and Pediatrics, Fujian Medical University, Fuzhou, China; ^2^Department of Radiology, Fujian Maternity and Child Health Hospital, College of Clinical Medicine for Obstetrics and Gynecology and Pediatrics, Fujian Medical University, Fuzhou, China; ^3^Department of Epidemiology and Health Statistics, School of Public Health, Fujian Medical University, Fuzhou, China

**Keywords:** fetus, echocardiography, double aortic arch, prenatal diagnosis, postnatal analysis

## Abstract

**Objective:**

This study aims to analyze the value of prenatal ultrasound in the screening, diagnosis, and treatment of double aortic arch (DAA) malformations.

**Methods:**

A retrospective analysis was conducted on 31 fetal cases with double aortic arch anomalies over a 12-year period from June 1, 2011 to June 1, 2023. The assessment included combined measurements of fetal tracheal internal diameter Z-score and DAA pinch angle, along with ultrasonographic findings, associated anomalies, genetic abnormalities, postnatal CTA images, and long-term postnatal outcomes.

**Results:**

Of the 31 fetal double aortic arch cases, 15 were right aortic arch dominant, 2 were left aortic arch dominant, and 14 had a balanced double arch. Genetic testing was performed on 19 cases, revealing abnormalities in 2 cases, including one Turner syndrome, and one carrier of ichthyosis gene with heterozygous deletion. Out of the total cases, 29 were delivered, and 2 cases were terminated. Prenatal diagnosis accurately identified 29 cases (29/31, 93.5%), which was confirmed by postnatal pathological anatomy, echocardiography, surgery or CTA. Fetal tracheal internal diameter Z-scores were significantly smaller in the symptomatic group than in the asymptomatic group (−1.27 ± 0.49 vs −0.68 ± 0.60, *P* = 0.018). The area under the curve was 0.776 (95% confidence interval, 0.593–0.960) using a tracheal internal diameter z-score cutoff of −0.73 with a sensitivity of 90% and specificity of 64.7%. The double arch pinch angle was significantly smaller in the symptomatic group than in the asymptomatic group [52.50° (38.25° to 59.00°) vs 60.00° (53.50° to 70.50°), *P* = 0.035]. The area under the curve was 0.744 (95% confidence interval, 0.554–0.935), and the sensitivity for determining the presence or absence of symptoms was 90% when the cutoff value was 62.5°, with a specificity of 47.1%. Fifteen cases opted for surgery with favorable surgical outcome.

**Conclusion:**

Prenatal echocardiography demonstrates good diagnostic efficacy for fetal double aortic arch. It is also essential to detect the presence of other underlying intra- and extracardiac malformations and genetic abnormalities. There is a significant difference in prenatal tracheal internal diameter Z-scores and double arch pinch angle between asymptomatic and symptomatic DAA infants. Symptomatic infants require early surgery, while asymptomatic infants should be monitored.

## 1 Introduction

Double aortic arch (DAA) represents a rare congenital heart disease, distinguished by the division of the ascending aorta into right and left branches anterior to the trachea and esophagus. These branches encircle both structures before converging to form the descending aorta. This condition typically arises from abnormal embryonic development of the fourth pair of aortic arches (1). The incidence of DAA is approximately 0.005–0.007%, accounting for 33–75% of fetal vascular ring malformations (2, 3). According to the classification proposed by Backer and colleagues, DAA is categorized into three types: right arch dominant, left arch dominant and double arch balanced. The right arch dominant type is the most common, representing about 75% of all cases (4, 5). Approximately 16.6% of DAA cases may be associated with intracardiac malformations, such as tetralogy of Fallot, transposition of the great arteries, and ventricular septal defects, while 24% may be linked to chromosomal abnormalities, including 22q11 deletion syndrome (6, 7).

Most patients affected by DAA present with symptoms early in infancy. However, DAA can also be asymptomatic and found incidentally later in life through chest imaging or echocardiography. When the double aortic arch compresses the trachea and esophagus, it can lead to a variety of symptoms, including dysphagia, asthma, and dyspnea (4, 8). Repeated episodes of wheezing may affect bronchopulmonary development, potentially causing bronchial dysplasia and, in severe cases, respiratory failure or death. Studies have shown that children with DAA are more likely to present earlier with symptoms and experience residual respiratory morbidity post-operation compared to those with a right aortic arch (RAA). Some surgical centers advocate for universal early surgery in these cases. Prenatal diagnosis aids perinatal management by enabling parental education, early identification of clinical features, and timely assessment and treatment. Additionally, it has been associated with reduced residual respiratory morbidity after surgical repair, especially in children with DAA. Early surgical intervention is crucial to prevent long-term morbidity. Therefore, accurate prenatal diagnosis of DAA is crucial for neonatal prognosis (9–14).

To improve the accuracy of prenatal diagnosis of DAA, several new ultrasound techniques and indices have been proposed. High-resolution flow (HD-Flow) and time-space composite imaging (STIC) are among the methods used for diagnosis (15–18). Furthermore, the fetal tracheal internal diameter Z-score has been employed to quantitatively assess tracheal compression due to DAA (15). Additionally, differentiating the different pinch angles of DAA from those of the mirrored right aortic arch (RAA-MB) can improve the diagnostic accuracy of these vascular structures (19). Although multiple tests are available, making a comprehensive and accurate prenatal diagnosis of DAA remains challenging.

We conducted a retrospective analysis to study the prenatal ultrasound features of fetal DAA in conjunction with postnatal computed tomography angiography (CTA) features of CTA characteristics, associated abnormalities, and clinical outcomes. We also analyzed the Z-score of fetal tracheal internal diameter and measurements of the DAA pinch angle. The aim was to refine the prenatal diagnosis of DAA and to provide support for perinatal management.

## 2 Materials and methods

### 2.1 Study population

**FIGURE 1:**
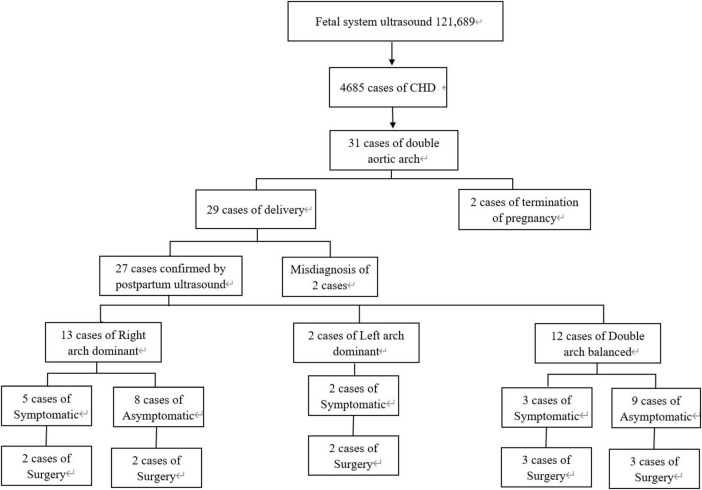
Flowchart of this retrospective study.

Between June 1, 2011 and June 1, 2023, a total of 121,689 fetuses underwent systematic ultrasonography, with 73,719 fetuses examined at 11 to 13+6 weeks. There were 4,685 cases of congenital heart disease diagnosed (Figure 1). A total of 31 fetuses with DAA were confirmed pathologically, by postnatal imaging, or by surgery. The mean maternal age was 28.32 ± 4.65 (range 18–39) years, and the mean gestational age 25.57 ± 3.68 (range 13–37) weeks (Table 1). All study subjects were followed up by telephone at 3 and 6 months postpartum, and then once a year thereafter. The study was approved by the Ethics Committee of Fujian Maternity and Child Health Hospital, Fujian Medical University (2014FY110700), and informed consent was obtained from the families. In cases where pregnancy was terminated, families signed the informed consent for autopsy. According to Backer’s classification criteria, DAA was classified as right arch dominant, left arch dominant, and double arch balanced. Based on this classification, DAA not associated with other abnormalities was defined as simple DA, while DAA combined with other abnormalities was defined as non-simple DAA in this study. The associated abnormalities included: soft index abnormalities, intracardiac abnormalities, and extracardiac abnormalities. Postnatal children were categorized into the symptomatic group if they had the following symptoms: respiratory symptoms, such as wheezing, coughing, dyspnea, or choking; and esophageal symptoms, such as difficulty feeding or swallowing. Those without these symptoms were included in the asymptomatic group.

**TABLE 1:** Baseline characteristics of fetuses with DAA.

Characteristics	Values (*n* = 29)
Maternal age (y)	28.59 ± 4.36
Gestational age at diagnosis (wk)	25.03 ± 4.28
**Backer’s classification**	
Right arch dominant	13
Left arch dominant	2
Double arch balanced	14
Isolated DAA	23
Non-isolation DAA	6
Intracardiac anomalies	3
Extracardiac anomalies	1
USM anomalies	2
Normal genetic detection	17
Abnormal genetic detection	2
**Clinical outcome**	
Termination of pregnancies	2
Live births	27
Symptomatic	10
Asymptomatic	17
Surgery	14

### 2.2 Fetal echocardiography

Utilizing advanced Color Doppler ultrasound diagnostic equipment such as the GE Voluson S8, E8, E10, and Samsung W10 and Philips EPIQ 7C, fetal cardiac screening are conducted in accordance with the stringent International Society of Ultrasound in Gynecology and Obstetrics (ISUOG) guidelines (20). The transducer frequency is configured to range between 4.0–8.0 MHz. The nine-sectional methodology is employed for comprehensive of the fetal heart (21). In instances where a DAA is suspected, the focus intensifies on acquiring detailed views of the three-vessel series, which encompasses the three-vessel view, the three-vessel-tracheal view, the coronary view of the aortic arch, and the long-axis view of the aortic arch. These specific planes are essential for the determination of the aortic branches and arterial ducts. If the two arches are not on the same plane during the standard three-vessel-tracheal view, a subtle oblique adjustment of the probe becomes necessary to affect their simultaneous visualization.

**FIGURE 2:**
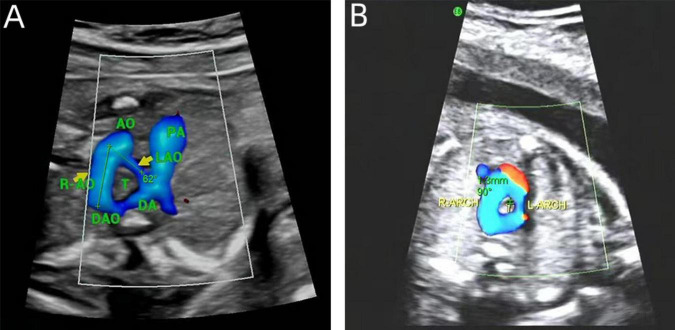
Measurements on prenatal ultrasound images. **(A)** DAA pinch angle; **(B)** the tracheal internal diameter Z-value.

In DAA fetuses, the internal diameter of the trachea was measured during the real-time examination or by reviewing a still image or a video from the initial diagnosis of DAA. These measurements were precise, taken only when the three-vessel-tracheal view distinctly presented the double aortic arches encircling the trachea. We used validated measurement tools and software for image analysis, and two experienced physicians independently conducted the measurements to ensure consistency and repeatability. Each measurement was repeated three times and an average was calculated to ensure accuracy. Subsequently, calculations were performed according to the Z-value formula proposed by Yin et al. (15) (1) predicted tracheal internal diameter = (0.147 × GA) - 1.423; (2) predicted SD = $\sqrt{\pi }/2$ × 0.029 × GA; (3) Z-value = (observed tracheal ID - predicted tracheal ID)/predicted SD. The right aortic arch is connected to the central axis of the right aortic arch as line A, and the left aortic arch is connected to the central axis of the left aortic arch as line P. The two lines intersect at point O to obtain ∠AOP, which is the angle between the right aortic arch and the left aortic arch in the DAA (Figure 2).

### 2.3 Neonatal imaging

Postpartum echocardiography was performed using the Philips EPIQ 7C and IE Elite diagnostic ultrasound machine, with the probe frequency set at 3.0–8.0 MHz, in accordance with the American Society of Echocardiography (ASE) Guidelines for Pediatric Echocardiography (22). A comprehensive scan of the heart was performed by segmental analysis to examine the origin, internal diameter, and blood flow of the aorta and its branches. In cases where a DAA is suspected, the ultrasound focus is directed toward the long and short axis views of the aortic arch in the suprasternal fossa.

Utilizing the GE Revolution 256-row CT scanner, a child was sedated and placed in a supine position. The scanning ranges from the root of the neck to 4 cm below the diaphragm. A routine plain scanning, was initially performed, followed by a CTA examination. The scanning parameters were set at 80∼100 KV, 110∼300 mA, and a volumetric scanning technique (with the ability to reconstruct images with a layer thickness of 0.625 mm). The layer thickness was maintained at 3 mm, and a high-pressure syringe automatically pushed at a Flow rate 1.0∼1.5 mL/s. The contrast agent with iopromide (370 mgI/mL) was administrated with a dose of 1.0∼1.5 mL/kg), scanning delay time was determined using the threshold method, with the ROI placed in the root of the aorta or pulmonary artery trunk, and the threshold value set at 100HU. When the contrast agent reached this threshold, the scanning was automatically triggered. The CT raw reconstruction thin layer data were transferred to Advantage Windows 4.6 workstation for multiplanar reconstruction (MPR), CT Volume Rendering (CTVR), Minimum Density Projection (MIP) reconstruction, and three-dimensional (3D) reconstruction of the aorta (23). In case of Where a suspected DAA was detected, key areas of observation were located in the ascending aorta and the aortic arch.

### 2.4 Genetic examination

All pregnant women who received a diagnosis of DAA through ultrasound scanning were provided with comprehensive counseling about the diagnosis and treatment options available. Some women underwent further chromosomal karyotyping and single nucleotide polymorphism microarray (SNP microarray) analysis of fetal amniotic fluid or umbilical cord blood to screen for any underlying genetic their fetus.

### 2.5 Statistical analysis

SPSS 26.0 software was used to analyze the data statistically. The Measurement data were expressed as mean standard deviation (SD). For normally distributed data, a T-test was employed to compare the difference between two groups. In instances where data were not normally distributed, the Mann-Whitney U test was utilized, and the data was presented using median and quartile [M(IQR)]. To analyze the diagnostic efficacy of new indexes, Receiver Operating Characteristic (ROC) curves were used. These curves allowed for the assessment of the performance of binary classification systems, providing an area under the curve (AUC) that indicates the accuracy of the test. A two-sided test was performed to determine statistical significance, with a P value less than 0.05 considered to indicate a significant difference. Thus, if the P-value was less than 0.05, it was concluded that there was a significant difference between the groups compared.

## 3 Results

There were 31 DAA cases, including 2 left arch dominant (6.5%), 15 right arch dominant (48.39%), 14 double arch balanced (45.16%). All 31 cases had left-position arterial conduits. There were 24 cases (77.4%) in the simple DAA group and 7 cases (22.6%) in the non-simple DAA group. Among the Non-simple DAA cases, 2 fetuses were confirmed by autopsy after termination of pregnancy. A total of 29 live birth were recorded, with a live birth rate of 93.5% (29/31). Postnatal echocardiography, CTA, or surgery confirmed the diagnostic accuracy of 93.5% (29/31) for prenatal ultrasonography. Two cases were misdiagnosed or missed prenatally, for a misdiagnosis rate of 6.5% (2/31).

### 3.1 Prenatal echocardiographic features of the double aortic arch

**FIGURE 3:**
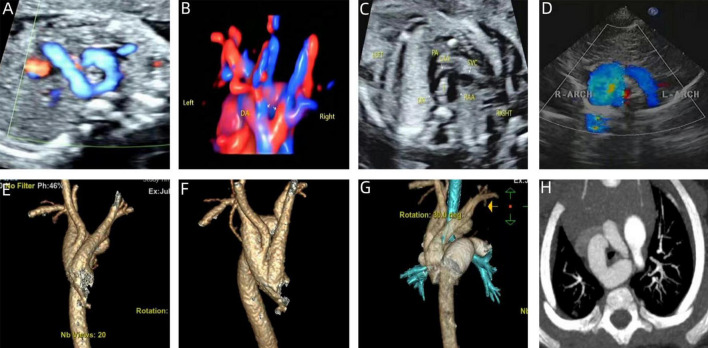
Prenatal-postpartum imaging of right arch dominant. **(A–C)** Three-vessel tracheal section with an “O” shape around the trachea and a thin left aortic arch; **(D)** echocardiographic manifestations of right arch dominance in the neonatal period; **(E–G)** three-dimensional reconstruction of CT in the neonatal period; **(H)** CTA image in the neonatal period with left arch atresia.

**FIGURE 4:**
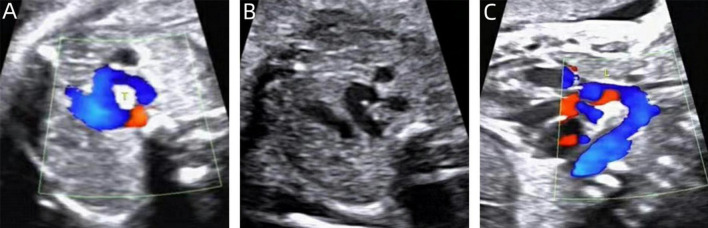
Two-dimensional gray-scale ultrasound and Color Doppler of left arch dominant. **(A–B)** Three-vessel tracheal view showing a left arch with a larger internal diameter than the right arch and a left arterial conduit, forming a complete “O” shaped vascular ring. **(C)** Color Doppler in long-axis view of the aortic arch.

**FIGURE 5:**
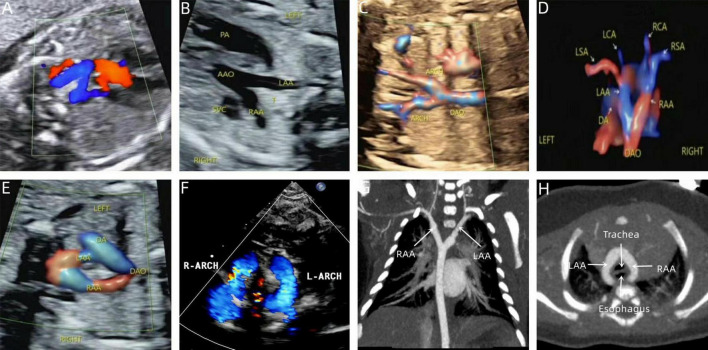
Prenatal-postpartum imaging of double arch balanced. **(A)** Three-vessel tracheal view showing the left and right aortic arches forming a “Z” shape with the left arterial conduit; **(B,C)** The ascending aorta divides into left and right aortic arches in a “Y” shape. **(D,E)** STIC and color Doppler showing left and right aortic arch forming an “O” shaped vascular ring encircling the trachea. **(F)** Neonatal echocardiography; **(G,H)** CTA shows that the left and right aortic arches are the same size.

The echocardiographic features of DAA in the fetal stage. Include the characteristic “Y” shaped appearance of the ascending aorta, which branches into the left and right aortic arches. These arches give rise to the common carotid artery and the subclavian artery, respectively. The twin arches then encircle the trachea and esophagus, forming an “O”-shaped ring of blood vessels. These vessels converge at the descending aorta, which is located posterior to the trachea. Color Doppler flowmetry revealed that the double arches were connected to the left arterial conduit in a pattern resembling either a “6” or a “9” (Figures 3–5). Among the cases, there were two instances of left arch dominance, both of which were classified as simple DAA. Of the 15 cases with right arch dominance, 11 were simple DAA and 4 were non-simple, including 1 with NT thickening detected by ultrasonography in early pregnancy, double aortic arches were diagnosed in mid-gestation, one where the left kidney was not detected prenatally and was later found to be associated with vaginal diagonal septal syndrome postnatally, one case where one of the twins had ventricular septal defects and pulmonary artery stenosis, and one case with a left innominate vein under the arch.

There were 14 cases of double arch balanced, with 11 being simple DAA and the 3 non-simple, including one where ultrasonography in early pregnancy revealed NT thickening and double aortic arch, with the diagnosis of double aortic arch confirmed in mid-pregnancy, one case was confirmed in mid-pregnancy to have double aortic arch combined with pulmonary atresia with ventricular septal defect (PAVSD), and one case of the left innominate vein under the arch (Table 2).

**TABLE 2:** Prognosis of 31 cases of fetal double aortic arch combined with intra- and extracardiac anomalies.

	Number	Intra- and extracardiac anomalies	Outcome		
			TOP	Parturition	Surgery
Left arch dominant	2	Null	0	2	2
Right arch dominant	15	NTT, LIV, Absence of left kidney	0	15	7
Double arch balanced	14	NTT, PAVSD, LIV, COA	2	12	6
Total	31		2	29	15

NTT, Nuchal translucency thickening; PAVSD, Pulmonary atresia with ventricular septal defect; LIV, left innominate vein; COA, Coarctation of the aorta; TOP, Termination of pregnancies.

**FIGURE 6:**
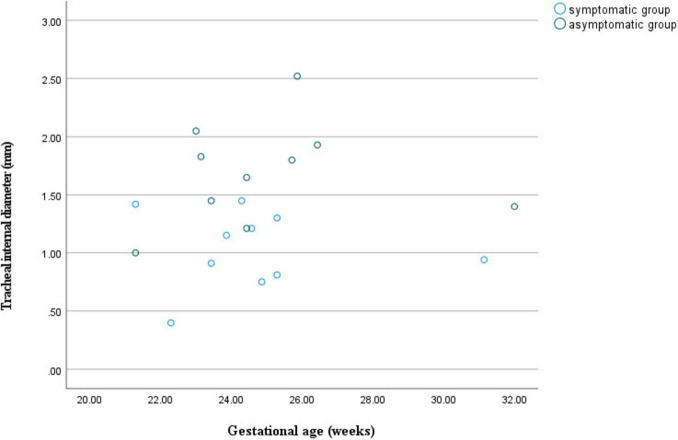
Scatter plot of tracheal internal diameter in relation to gestational age for double aortic arch (with or without symptoms).

**FIGURE 7:**
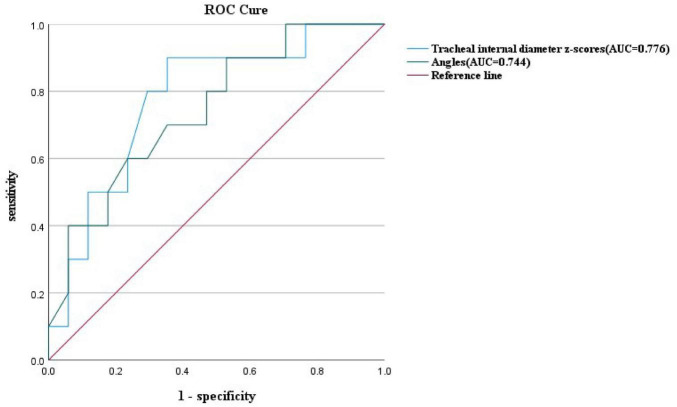
ROC curves and their predictions for the double aortic arch tracheal internal diameter z-scores cutoffs and double aortic arch pinch angle cutoffs.

At similar gestational ages, the tracheal internal diameter in the symptomatic group is significantly smaller than in the asymptomatic group (Figure 6). Compared with the asymptomatic group, the Z-value of the tracheal internal diameter was significantly reduced in the symptomatic group (−1.27 ± 0.49 vs −0.68 ± 0.60, *P* = 0.018). The area under the curve was 0.776 (95% confidence interval, 0.593–0.960) using a cutoff value of −0.73 for the tracheal internal diameter z-value, with a sensitivity of 90% and a specificity of 64.7%. Additionally, compared with the asymptomatic group, the double arch angle was significantly reduced in the symptomatic group [52.50° (38.25° to 59.00°) vs 60.00° (53.50° to 70.50°), *P* = 0.035]. The area under the curve was 0.744 (95% confidence interval, 0.554–0.935), the cutoff value was 62.5°, and the sensitivity and specificity for determining the presence or absence of symptoms were 90 and 47.1%, respectively (Figure 7 and Table 3).

**TABLE 3:** Comparisons of the clinical characteristics and tracheal internal diameter z scores between the asymptomatic and symptomatic groups in infants with DAA.

	Asymptomatic (*n* = 10)	Symptomatic (*n* = 17)	*p-*value
Maternal age (y)	29.0 (27.0–32.3)	28.0 (24.5–32.0)	0.19
Gestational age at diagnosis (wk)	24.4 (23.1–25.3)	24.4 (23.5–27.6)	0.54
**Type**			
Right arch dominant	5 (50.0%)	8 (47.1%)	
Left arch dominant	2 (20.0%)	0	
Double arch balanced	3 (30.0%)	9 (52.9%)	
Isolated DAA	9	14	
Non-isolation DAA	1	3	
Intracardiac anomalies	1	1	
Extracardiac anomalies	0	1	
USM anomalies	0	1	
**Surgery**			
YES	9	5	
NO	1	12	
Tracheal internal diameter (mm)	1.05 (0.80–1.33)	1.65 (1.31–2.09)	<0.001
Tracheal internal diameter z-scores	−1.27 ± 0.49	−0.68 ± 0.60	0.02
Angles	52.50° (38.25° to 59.00°)	60.00° (53.50° to 70.50°)	0.04

### 3.2 Postpartum echocardiographic and CTA findings in double aortic arch

**FIGURE 8:**
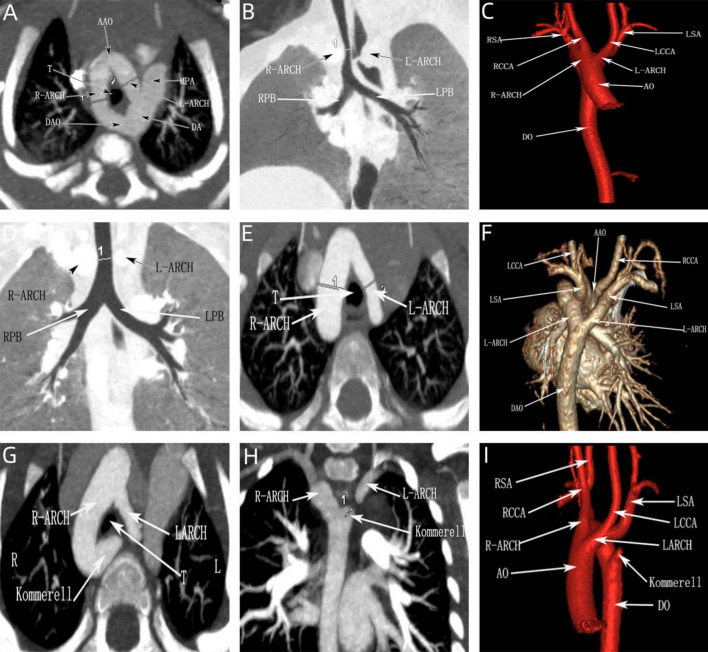
CTA of the double aortic arch in the neonatal period. **(A–C)** Double aortic arch compression of the trachea; **(D–F)** Double aortic arch without tracheal compression; **(G–I)** Double aortic arch (one arch dissected) with Kommerell diverticulum formation.

All the postpartum children were examined by echocardiography, eight cases that were diagnosed as right arch dominant by prenatal ultrasound were found to have left arch atresia by postnatal repeat ultrasound, four of which were confirmed by CTA. Sixteen children underwent CTA, of which 12 cases were found to have tracheal stenosis, including six cases of right arch dominant, one case of left arch dominant, and five cases of equilibrium, including one case of right arch dominant misdiagnosed prenatally as a right-sided aortic arch. In addition, in three cases, the formation of Kommerell’s diverticulum was not detected during prenatal ultrasonography, but this lesion was detected postnatally by CTA, and all of them showed significant stenosis of the trachea, two of which were accompanied by left arch atresia, and all of them showed significant respiratory symptoms (Figure 8). This stenosis is primarily due to external compression by the aortic arches, resulting in secondary compression, rather than intrinsic tracheal narrowing.

### 3.3 Chromosomal findings in double aortic arch

In term of chromosomal findings, out of 31 cases of DAA, 19 underwent genetic testing, with abnormalities hound in 2 cases (10.5%). All 14 cases of simple DAA underwent genetic testing showed no abnormality while 2 out of 5 cases of non-simple DAA were abnormal, one being Turner’s syndrome and the other being an ichthyosiform disease gene carrier combined with a heterozygous deletion, both associated with NT thickening. Fisher’s exact probability method revealed no statistically significant difference between simple and non-simple types concerning genetic abnormalities (*P* = 0.06) (Table 4).

**TABLE 4:** Comparison of incidence of chromosome abnormality between Isolated DAA and non-isolation DAA.

	Total	Abnormal	Ratio	*P*-value
Isolated DAA	14	0	0%	0.06%
Non-isolation DAA	5	2	40.00%	

### 3.4 Clinical manifestations

Clinical symptoms such as asthma, dysphagia, and choking occurred in 12 children due to significant tracheal compression, with an incidence of 41.4% (12/29). A total of 15 children [51.7% (15/29)] underwent surgical intervention, of which 10 were operated due to the development of clinical symptoms, representing 66.7% (10/15) of the operated children. The other 5 children were operated based on the family’s concerns about the long-term prognosis. After surgical treatment, all the children’s symptoms improved, and postoperative follow-up showed that they were in good health with no significant abnormalities on repeat echocardiograms. Of the remaining 14 children who were not operated on, only one had mild symptoms of shortness of breath, and the cardiac surgeon recommended temporary observation (Table 5).

**TABLE 5:** Details of the 29 fetuses with double aortic arch who were born alive.

Case	Type	Symptoms	Time to symptoms	Surgery	Time of surgery
1	Left	Cough	17 months	Yes	23 months
2	Left	Cough	3 months	Yes	6 months
3	Right	Cough	15 months	No	
4	Right	No		No	
5	Right	Cough	1 month	Yes	2 months
6	Right	Cough	47 months	Yes	54 months
7	Right	Cough	3 months	Yes	5 months
8	Right	No		No	
9	Right	Cough	17 months	Yes	22 months
10	Right	No		No	
11	Right	No		No	
12	Right	No		Yes	5 months
13	Right	No		No	
14	Right	No		No	
15	Right	No		No	
16	Balanced	No		No	
17	Balanced	pneumonia	2 months	Yes	8 months
18	Balanced	No		No	
19	Balanced	pneumonia	5 months	Yes	8 months
20	Balanced	No		Yes	24 months
21	Balanced	No		Yes	3 months
22	Balanced	No		Yes	3 months
23	Balanced	No		No	
24	Balanced	Cough	1 month	Yes	2 months
25	Balanced	No		No	
26	Balanced	No		Yes	6 months
27	Balanced	Cough	9 months	No	
28	Balanced	Cough	15 months	No	
29	Balanced	No		No	

## 4 Discussion

In recent years, significant progress has been made in prenatal and postnatal studies on DAA. In the realm of prenatal diagnosis, advancements in fetal cardiac ultrasound technology have led to early identification of DAA. Moreover, the utilization of three-dimensional ultrasound and color doppler ultrasound has refined hemodynamic assessment. for postnatal diagnosis, transthoracic and transesophageal echocardiography severe as primary diagnostic tools, allowing confirmation of the presence of a DAA, as well as the evaluation of vascular anatomy, size, and interrelationships. In some cases, more sophisticated imaging techniques such as MRI or CT may be required to obtain detailed information about the vascular structure. Despite these technical advances, challenges remain in the diagnosis of DAA, particularly in early cases where accurate diagnosis may not be feasible. Furthermore, while surgery is the mainstay of treatment for DAA, the choice of surgical approach and timing continues to be a subject of refinement.

In this study, the right arch dominant type was the most common, with 15 cases (48.39%), which is generally consistent with the literature. However, it is noteworthy nothing that the proportion of double arch balanced type has increased as compared to previous studies. The observation that children with double arch balanced type tend to present earlier with symptoms such as shortness of breath and coughing in the neonatal period suggests that critically ill infants may not be diagnosed in time due to respiratory failure, potentially leading to an underestimation of the prevalence of this type. Therefore, in the integrated prenatal and postnatal management of DAA, a clear prenatal diagnosis and staging are particularly important. Additionally, it was noted that approximately 16.6% of DAA cases may be associated with intracardiac malformations such as tetralogy of Fallot, transposition of the great arteries and ventricular septal defects. In the present study, the percentage of combined intracardiac malformations was 12.9%, which is slightly lower than that reported in the literature, and this discrepancy may not adequately reflect the actual incidence of intracardiac malformations due to the small sample size.

We have found that although the majority of DAA can be successfully diagnosed through prenatal ultrasound, the diagnostic process remains challenging. In particular, the three-vessel-tracheal view, which is a key aspect of DAA diagnosis, is not always sufficient to confirm the condition on its own, this is particularly true in cases of left arch atresia, where typical vascular ring ultrasound manifestations may not be present due to interrupted blood flow. Therefore, the differential diagnosis between DAA from other vascular ring diseases such as pulmonary artery sling (PAS) and right aortic arch with mirror-image branches, is crucial. In this context, both the three-vessel-tracheal and three-vessel-pulmonary artery branch views are essential for differential diagnosis. For example, the key to the diagnosis of a pulmonary artery sling is that the three-vessel-pulmonary artery branching view fails to show the normal pulmonary artery bifurcation, but instead shows the left pulmonary artery originating from the right pulmonary artery and wrapping around the trachea. Conversely, identifying a right aortic arch with mirror-image branches hinges on observing whether the cephalic brachial artery is directly connected to the descending aorta. In addition, left ventricular outflow tract views of DAA may reveal changes in aortic bifurcation, which need to be differentiated from pulmonary artery originating from the ascending aorta (AOPA) and complete transposition of the great arteries (TGA). We also note that most of the double arches in DAA are unevenly developed, with approximately 75% being right arch dominant and generally located higher than the left arch. To reduce misdiagnosis, the number of branches of the cephalic and brachial arteries emanating from the aortic arch should be carefully identified during screening, and once two cephalic and brachial arteries are probed, high vigilance should be exercised for the presence of atresia of one side of the arches; moreover, the probe should be moved laterally in three-vessel-tracheal views to make both arches visible, to avoid missed diagnosis due to the high position of one side of the arches. Although studies have shown that the preoperative ultrasound detection rate of DAA is as high as 96% (24), some patients are still misdiagnosed or underdiagnosed in practice due to the lack of awareness of DAA by some sonographers or equipment limitations. Among the 31 fetuses with DAA in this study, 29 were born alive, of which 27 were compatible with prenatal ultrasound and 2 were confirmed by autopsy after induction of labor, resulting in a diagnostic accuracy of 93.5%. The misdiagnosed case was prenatally diagnosed with a right-sided aortic arch and underwent surgical treatment at 1 year and 10 months old due to the onset of cough and other symptoms. In contrast, the missed case had no detectable intra- or extracardiac malformations prenatally and was diagnosed with a double aortic arch postnatally. This condition was not surgically treated because the symptoms were mild. Research has demonstrated that the “Z” sign is the most predictive marker in fetal echocardiography (sensitivity: 100%, specificity: 81%) (25, 26). The “Z” sign is a common feature in fetal echocardiograms of double aortic arch, which helps to differentiate between a right aortic arch and a double aortic arch. The “6/9” sign is typically used to identify common variations of the aortic arch, whereas the “Z” sign is particularly helpful in identifying the complex condition of a double aortic arch. However, based on the data and case analyses we collected, we found the “6/9” sign to be more prominent and common in our fetal samples (Figures 4, 5).

DAA is a common type of intact vascular ring that often compresses the trachea or esophagus, causing infants to experience symptoms such as difficulty swallowing, asthma, and respiratory distress. In severe cases, infants may even develop respiratory failure, which constitutes a life-threatening condition. In this study, we used the tracheal internal diameter z-scores proposed by Yin et al. and found that there was a significant difference in the tracheal internal diameter z-scores of DAA fetuses in the symptomatic and asymptomatic groups, which not only agrees with the findings of Yin et al. but also further deepens our understanding of tracheal pressure in DAA fetuses, which may have an important impact on clinical diagnosis and treatment strategies. Repeatability is one of the basic principles in scientific research, and our study not only further validates the reliability and validity of the tracheal internal diameter z-scores, but also reduces the random error by having a larger sample size, thus making the results more credible. Recognizing and understanding neonatal respiratory symptoms caused by DAA is the key to this study, and by applying the tracheal internal diameter z-scores, physicians can predict the onset of these symptoms earlier and intervene in a timely manner, thus significantly improving the prognosis of the children and reducing the risk of associated complications.

In this study, by measuring the DAA pinch angle, we found that the double arch pinch angle in the symptomatic group was significantly smaller than that in the asymptomatic group, which is consistent with the findings of Han et al. (19). This study emphasizes the importance of prenatal ultrasound in predicting whether a child is exhibiting symptoms or not, and by measuring the double-arch pinch angle and comparing it with the cutoff value, we can arrive at a more accurate diagnosis. The experimental design differed from previous studies in that only the angle between the left and right arterial arches was measured and not compared with the RAA-MB, which provides a new perspective on this issue. Given the potential importance of dual aortic arch angle in the prediction of symptomatology in children, further studies are necessary. To further enrich the body of knowledge in this area, we plan to increase the sample size and further improve the measurement methods in future studies, which will help to validate our current findings and also hopefully reveal new insights and findings. In addition, by comparing the area under the ROC curve of the tracheal internal diameter Z-value and DAA pinch angle, we found that the tracheal internal diameter Z-value was more efficacious than DAA pinch angle in predicting symptoms (Figure 7). Therefore, when we are unable to measure both parameters, it is more important to prioritize the measurement of fetal tracheal internal diameter Z-value.

This study explored the association between DAA and genetic abnormalities, particularly chromosome 22q11 deletions. It was shown that the percentage of 22q11 deletions in patients with DAA is approximately 6%, which is lower than the 6.1–10% in fetuses with right aortic arch (27–29). In our study, only in the non-simple DAA group, 2 cases (10.5%) showed genetic abnormalities, but no 22q11 deletion was found (Table 4). Statistical analysis showed that there was no significant difference in the detection of genetic abnormalities between simplex and non-simplex DAA, a result that may be related to the small sample size. In addition, we found one case of ichthyosis gene carriage combined with heterozygous deletion and one case of Turner syndrome, which did not appear in previous related studies; therefore, further exploration of the association between DAA and genetic abnormalities is necessary. The present study also revealed the phenomenon of gender differences, i.e., DAA seems to be more common in males, which is not only reflected in our study (male-to-female ratio of 21:10), but is also consistent with the study of Helen Bornaun et al. (male-to-female ratio of 11:3). This finding suggests that sex chromosomes may play an important role in DAA occurrence and warrants further investigation. However, this study faced the limitation of a small sample size, which may affect the overall understanding of the relationship between DAA and genetic abnormalities. Future studies should expand the sample size and use gene editing technology and big data analysis to explore the genetic mechanism of DAA in depth, with the aim of deepening the understanding of DAA’s genetic factors.

Echocardiography is the routine postnatal diagnostic method for DAA, but it has limitations in identifying specific anatomical features (e.g., fibrous cords and tracheal compression), and air in the lungs may interfere with image quality. In contrast, CTA provides more comprehensive information, including vascular structures and spatial relationships with neighboring organs (especially the airway and esophagus), and is able to clearly show details of the compressed trachea, esophagus, and vascular rings (30, 31). There were 2 special cases of interest in this study, one of which was DAA with left arch atresia and the other was DAA with Kommerell diverticulum formation (Figures 3, 8). These two cases were misdiagnosed prenatally as right aortic arch and double aortic arch (right arch dominant type), respectively, but the correct diagnosis was obtained by CTA after birth. According to previous studies, Kommerell’s diverticulum (KD) is most commonly seen in patients with right aortic arch, whereas concomitant DAA is very rare, with only two case reports (32, 33). It has been suggested that DAA with Kommerell’s diverticulum is more likely to lead to compression of the esophagus or trachea, leading to symptoms, which is an important indication for surgical treatment. In patients with DAA, Kommerell’s diverticulectomy is safe and effective and also helps to prevent recurrence of symptoms after surgery (34, 35). The patient in this study, who presented with significant coughing symptoms in daily life, underwent surgery 1 year ago and is currently in good condition.

Fetal echocardiography is a reliable method for diagnosing fetal DAA. In particular, three-vessel-tracheal views and aortic arch coronal views are particularly important for screening. Aortic arch long-axis views help in the differential diagnosis of DAA. It is important to note that the prognosis of children with DAA depends not only on the DAA itself, but also on the presence and severity of other intra- and extracardiac malformations, as well as the presence of chromosomal abnormalities. For children with simple DAA or only other simple intra- and extracardiac malformations, surgical intervention usually yields satisfactory results, and induction of labor is generally not recommended. In symptomatic children, early surgical intervention usually yields favorable results. In children without symptoms of airway or esophageal compression, the condition can be monitored through long-term follow-up. In addition, close collaboration between cardiologists, geneticists, pediatricians, surgeons, and imaging physicians is essential for comprehensive management from prenatal to postnatal periods. The importance of long-term follow-up, including regular cardiac evaluations and psychosocial support when necessary, should not be overlooked, as these can have a profound impact on the quality of life of the child and his or her family.

## 5 Limitation

The limitations of the study were highlighted, including its single-center design with a small sample size. The genetic testing results did not conclusively determine the cause of fetal DAA, indicating that further investigation is necessary. there is a clear need for larger multicenter studies to validate the genetic findings and to provide a more comprehensive understanding of the genetic underpinnings of DAA. Additionally, the study raised questions about the management of asymptomatic DAA cases, particularly regarding whether these cases would require surgery in the future. this is an important issue that needs to be addressed by future research, as it directly impacts clinical decision-making and patient outcomes. longer-term follow-up studies are essential to understand the natural history of asymptomatic DAA and to determine the most appropriate interventions, if any, for these patients.

## 6 Conclusion

Prenatal ultrasonography is invaluable for diagnosing fetal DAA. The three-vessel view, three-vessel tracheal view and aortic arch long-axis view, and aortic arch coronal view are crucial for exam DAA. The prevalence of fetal DAA is low and most cases are simple DAA with a low association with chromosomal abnormalities. However, when non-simple DAA is detected, genetic testing to explore the correlation is necessary. There are significant differences in the tracheal internal diameter Z-value and double arch pinch angles between infants with asymptomatic and symptomatic DAA, and prenatal monitoring of both can provide important information for clinical management in the perinatal period. Postnatal CTA provides a more accurate picture of tracheal compression, which is essential for guiding the timing of surgery. The presence or absence of combined intra- and extracardiac abnormalities and the degree of tracheoesophageal compression can affect the prognosis of DAA. Therefore, when dealing with children with DAA, a comprehensive consideration of these factors is important to ensure optimal outcome and prognosis.

## Data Availability

The original contributions presented in this study are included in this article/supplementary material, further inquiries can be directed to the corresponding authors.

## References

[1] Edwards J. Vascular rings related to anomalies of the aortic arches. *Mod Concepts Cardiovasc Dis.* (1948) 17:1. 18873268

[2] Achiron R Rotstein Z Heggesh J Bronshtein M Zimand S Lipitz S Anomalies of the fetal aortic arch: A novel sonographic approach to in-utero diagnosis. *Ultrasound Obstet Gynecol.* (2002) 20:553–7. 10.1046/j.1469-0705.2002.00850.x 12493043

[3] Mogra R Kesby G Sholler G Hyett J. Identification and management of fetal isolated right-sided aortic arch in an unselected population. *Ultrasound Obstet Gynecol.* (2016) 48:739–43. 10.1002/uog.15892 26918379

[4] Shah R Mora B Bacha E Sena L Buonomo C Del Nido P The presentation and management of vascular rings: An otolaryngology perspective. *Int J Pediatr Otorhinolaryngol.* (2007) 71:57–62. 10.1016/j.ijporl.2006.08.025 17034866

[5] Backer C Mavroudis C. Congenital heart surgery nomenclature and database project: Vascular rings, tracheal stenosis, *Pectus excavatum*. *Ann Thorac Surg.* (2000) 69:S308–18. 10.1016/s0003-497501279-5 10798437

[6] Guo Q Kong Y Zeng S Zhou J Wang X Shang Q Fetal double aortic arch: Prenatal sonographic and postnatal computed tomography angiography features, associated abnormalities and clinical outcomes. *BMC Pregnancy Childbirth.* (2020) 20:614. 10.1186/s12884-020-03300-4 33046002 PMC7552480

[7] Hunter L Callaghan N Patel K Rinaldi L Bellsham-Revell H Sharland G. Prenatal echocardiographic diagnosis of double aortic arch. *Ultrasound Obstet Gynecol.* (2015) 45:483–5. 10.1002/uog.13408 24817195

[8] Ma G Li Z Li X Peng Y Du Z Jin L Congenital vascular rings: A rare cause of respiratory distress in infants and children. *Chin Med J (Engl).* (2007) 120:1408–12. 17825169

[9] Backer C Mongé M Popescu A Eltayeb O Rastatter J Rigsby C. Vascular rings. *Semin Pediatr Surg.* (2016) 25:165–75. 10.1053/j.sempedsurg.2016.02.009 27301603

[10] Phelan E Ryan S Rowley H. Vascular rings and slings: Interesting vascular anomalies. *J Laryngol Otol.* (2011) 125:1158–63. 10.1017/S0022215111001605 21854690

[11] Aly S Papneja K Mawad W Seed M Jaeggi E Yoo S. Prenatal diagnosis of vascular ring: Evaluation of fetal diagnosis and postnatal outcomes. *J Am Soc Echocardiogr.* (2022) 35:312–21. 10.1016/j.echo.2021.09.010 34600045

[12] Vigneswaran T Van Poppel M Griffiths B James P Jogeesvaran H Rahim Z Postnatal impact of a prenatally diagnosed double aortic arch. *Arch Dis Child.* (2021) 106:564–9. 10.1136/archdischild-2020-318946 33115711

[13] Stephens E Eltayeb O Kennedy C Rigsby C Rastatter J Carr M Influence of fetal diagnosis on management of vascular rings. *Ann Thorac Surg.* (2022) 113:630–6. 10.1016/j.athoracsur.2021.01.025 33524348

[14] Biermann D Holst T Hüners I Rickers C Kehl T Rüffer A Right aortic arch forming a true vascular ring: A clinical review. *Eur J Cardiothorac Surg.* (2021) 60:1014–21. 10.1093/ejcts/ezab225 33970211

[15] Yin X Liu Y Wu L Zheng Q Peng R Xie H. Evaluation of the trachea in fetuses with double aortic arch using prenatal ultrasound: A retrospective cohort study. *Am J Obstet Gynecol MFM.* (2023) 5:100759. 10.1016/j.ajogmf.2022.100759 36191891

[16] Inamura N Taniguchi T Takada N. The telediagnosis of double aortic arch using spatio-temporal image correlation. *Echocardiography.* (2021) 38:1081–3. 10.1111/echo.15092 34018634

[17] Li T Li Q Ma B Qi P Wang J Yang L. Prenatal diagnosis of complete vascular ring using high-definition flow render mode and spatiotemporal image correlation. *Echocardiography.* (2021) 38:488–92. 10.1111/echo.14919 33586787

[18] Wang Y Fan M Siddiqui F Wang M Sun W Sun X Strategies for accurate diagnosis of fetal aortic arch anomalies: Benefits of three-dimensional sonography with spatiotemporal image correlation and a novel algorithm for volume analysis. *J Am Soc Echocardiogr.* (2018) 31:1238–51. 10.1016/j.echo.2018.07.010 30146186

[19] Han J Zhang Y Gu X Liu X Sun L Zhao Y The differential diagnosis of double aortic arch and right aortic arch with mirror-image branches in the fetus: A potential novel method. *Pediatr Cardiol.* (2021) 42:1405–9. 10.1007/s00246-021-02625-x 34258648

[20] International Society of Ultrasound in Obstetrics and Gynecology None, Carvalho JS Allan LD Chaoui R Copel JA DeVore GR ISUOG practice guidelines (updated): Sonographic screening examination of the fetal heart. *Ultrasound Obstet Gynecol.* (2013) 41:348–59. 10.1002/uog.12403 23460196

[21] Ling W Weng Z Qiu X Ma H Wu Q Liu M The value of nine-section segmental analysis in the diagnosis of complex congenital heart disease in the foetus. *Fujian Med J.* (2020) 42:9–13.

[22] Lai W Geva T Shirali G Frommelt P Humes R Brook M Guidelines and standards for performance of a pediatric echocardiogram: A report from the task force of the pediatric council of the American society of echocardiography. *J Am Soc Echocardiogr.* (2006) 19:1413–30. 10.1016/j.echo.2006.09.001 17138024

[23] Huang Q Ling W Wu Q Guo S Dang T Ma H Anomalous origin of the fetal pulmonary artery. *Front Pediatr.* (2023) 11:1204070. 10.3389/fped.2023.1204070 37456564 PMC10338927

[24] Bromley B Estroff J Sanders S Parad R Roberts D Frigoletto F Fetal echocardiography: Accuracy and limitations in a population at high and low risk for heart defects. *Am J Obstet Gynecol.* (1992) 166:1473–81. 10.1016/0002-937891622-h 1595802

[25] Van Poppel M Zidere V Simpson J Vigneswaran T. Fetal echocardiographic markers to differentiate between a right and double aortic arch. *Prenat Diagn.* (2022) 42:419–27. 10.1002/pd.6104 35060138

[26] Van Poppel M Pushparajah K Lloyd D Razavi R Speggiorin S Nyman A Insights from fetal cardiac magnetic resonance imaging in double aortic arch. *Ultrasound Obstet Gynecol.* (2020) 56:636–9. 10.1002/uog.22110 32484274

[27] D’Antonio F Khalil A Zidere V Carvalho J. Fetuses with right aortic arch: A multicenter cohort study and meta-analysis. *Ultrasound Obstet Gynecol.* (2016) 47:423–32. 10.1002/uog.15805 26643657

[28] Berg C Bender F Soukup M Geipel A Axt-Fliedner R Breuer J Right aortic arch detected in fetal life. *Ultrasound Obstet Gynecol.* (2006) 28:882–9. 10.1002/uog.3883 17086578

[29] Miranda J Callaghan N Miller O Simpson J Sharland G. Right aortic arch diagnosed antenatally: Associations and outcome in 98 fetuses. *Heart.* (2014) 100:54–9. 10.1136/heartjnl-2013-304860 24192976

[30] Kellenberger C. Aortic arch malformations. *Pediatr Radiol.* (2010) 40:876–84. 10.1007/s00247-010-1607-9 20354848

[31] Yoshimura N Fukahara K Yamashita A Doi T Yamashita S Homma T Congenital vascular ring. *Surg Today.* (2020) 50:1151–8. 10.1007/s00595-019-01907-5 31676999

[32] Kleine P Balci M Moritz A. Primary complete repair of partial double aortic arch and Kommerell diverticulum. *Ann Thorac Surg.* (2011) 91:627–9. 10.1016/j.athoracsur.2010.06.060 21256339

[33] Sierra-Galan L Shveid-Gerson D Gomez-Garza G Rey-Rodriguez A. Double incomplete aortic arch and Kommerell’s Diverticulum as a cause of chronic cough. *Arch Cardiol Mex.* (2015) 85:158–60. 10.1016/j.acmx.2014.12.009 25700574

[34] Bhatt T Muralidharan C Singh G Jain N. Kommerell’s diverticulum: A rare aortic arch anomaly. *Med J Armed Forces India.* (2016) 72(Suppl. 1):S80–3. 10.1016/j.mjafi.2016.09.003 28050078 PMC5192231

[35] Backer C Bharadwaj S Eltayeb O Forbess J Popescu A Mongé M. Double aortic arch with Kommerell diverticulum. *Ann Thorac Surg.* (2019) 108:161–6. 10.1016/j.athoracsur.2019.01.062 30849335

